# Blood pressure reduction and clinical outcomes with angiotensin-converting enzyme inhibitors and angiotensin II receptor blockers: protocol for a systematic review and meta-regression analysis

**DOI:** 10.1186/s13643-018-0779-5

**Published:** 2018-08-25

**Authors:** Jonathan A. Batty, Mengyao Tang, Marlous Hall, Roberto Ferrari, Martin H. Strauss, Alistair S. Hall

**Affiliations:** 1 Medical Research Council Bioinformatics Centre, Leeds Institute of Cardiovascular and Metabolic Medicine, University of Leeds, Leeds, UK; 2 Department of Cardiology, Leeds General Infirmary, Great George Street, Leeds, UK; 3 Department of Medicine, Vanderbilt University Medical Center, Nashville, TN USA; 4 Centro Cardiologico Universitario e and LTTA Centre, University of Ferrara, Ferrara, Italy; 5 Maria Cecilia Hospital, GVM Care and Research, ES Health Science Foundation, Cotignola, RA Italy; 6 Department of Medicine, University of Toronto, Toronto, Canada; 7 Leeds General Infirmary Old Site, Great George Street, Leeds, LS1 3EX UK

**Keywords:** Angiotensin-converting enzyme inhibitors, Angiotensin receptor blockers, Hypertension, Blood pressure, Heart failure, Myocardial infarction, Mortality, Clinical outcomes

## Abstract

**Background:**

Angiotensin-converting enzyme inhibitors (ACEis) and angiotensin II receptor blockers (ARBs) efficaciously reduce systolic blood pressure (BP), a well-established risk factor for myocardial infarction (MI). Both inhibit the renin-angiotensin system, albeit through different mechanisms, and produce similar reductions in BP. However, in parallel meta-analyses of ACEi and ARB trials, ACEis reduce risk of MI whereas ARBs do not—a phenomenon described as the ‘ARB-MI paradox’. In addition, ACEis reduce all-cause mortality, whereas ARBs do not, which appears to be independent of BP lowering. The divergent cardiovascular effects of ACE inhibitors and ARBs, despite similar BP reductions, are counter-intuitive. This systematic review aims to ascertain the extent to which clinical outcomes in randomised trials of ACEi and ARBs are attributable to reductions in systolic BP.

**Methods:**

A comprehensive search of bibliographic databases will be performed to identify all randomised studies of agents of the ACEi and ARB class. Placebo and active comparator-controlled studies that report clinical outcomes, with greater than 500 person-years of follow-up in each study arm, will be included. Two independent reviewers will screen study records against a priori-defined eligibility criteria and perform data extraction. The Cochrane Risk of Bias Tool will be applied to all included studies. Studies retracted subsequent to initial publication will be excluded. Primary outcomes of interest include MI and all-cause mortality; secondary outcomes include stroke, heart failure, revascularisation and cardiovascular mortality. Meta-regression will be performed, evaluating the relationship between attained reduction in systolic BP and relative risk of each outcome, stratified by drug class. Where a BP-dependent effect exists (two-tailed *p* value < 0.05), relative risks, standardised per 10 mmHg difference in BP, will be reported for each study outcome. Publication bias will be examined using Funnel plots, and calculation of Egger’s statistic.

**Discussion:**

This systematic review will provide a detailed synthesis of evidence regarding the relationship between BP reduction and clinical outcomes with ACEi and ARBs. Greater understanding of the dependency of the effect of each class on BP reduction will advance insight into the nature of the ARB-MI paradox and guide the future usage of these agents.

**Systematic review registration:**

PROSPERO CRD42017072988

**Electronic supplementary material:**

The online version of this article (10.1186/s13643-018-0779-5) contains supplementary material, which is available to authorized users.

## Introduction

Elevated systolic blood pressure (BP) is a well-established risk factor for myocardial infarction (MI), stroke, heart failure, and death [1–4]. This risk may be ameliorated with antihypertensive treatment: a reduction in systolic BP of 10 mmHg translates into a 17% reduction in the incidence of coronary artery disease, a 27% reduction in stroke, a 28% reduction in heart failure, and a 13% reduction in all-cause mortality [5]. Pharmacological inhibition of the renin-angiotensin-aldosterone system has been robustly demonstrated to reduce blood pressure by numerous randomised clinical trials, meta-analyses and observational studies [6, 7]. However, controversy persists over the comparative safety and efficacy of angiotensin-converting enzyme inhibitors (ACEis) and angiotensin II receptor blockers (ARBs), particularly with regard to myocardial infarction (MI) [8, 9]. Despite robust evidence that ARBs reduce BP, stroke and heart failure, no study has demonstrated a significant, protective impact of ARBs for MI, cardiovascular or all-cause mortality—endpoints reduced by ACEis in multiple trials [7, 10]. Indeed, in several studies, a potential ARB-associated increased risk of MI has been suggested [11, 12]. That ARBs reduce BP—but not MI—has been described as the ‘ARB-MI paradox’ [13].

Several putative biological hypotheses for this paradox have been advanced. Firstly, ARBs may have a pharmacological action that increases the risk of MI, independent of, and partially masked by their blood pressure-lowering effects [13]. ARBs induce selective antagonism of the angiotensin II type I (AT_1_) receptor, reducing downstream aldosterone secretion, salt and water retention. However, AT_1_ inhibition uncouples the angiotensin II negative feedback loop, leading to marked counterregulatory upregulation; angiotensin II levels increase 2- to 3-fold from baseline [14]. Putatively, this could lead to a greater risk of MI via increased stimulation of angiotensin II type II (AT_2_) receptors, which are overexpressed in atheromatous plaques, and may promote plaque vulnerability [15]. Other plausible adverse effects of AT_1_ blockade include increases in plasminogen activator inhibitor-1 (PAI-1) and reductions in bradykinin. Conversely, ACEis suppress angiotensin II synthesis and inhibit the breakdown of bradykinin; yielding synergistic cardioprotective effects.

Some authors have dismissed the validity of the ARB-MI paradox; attributing the lack of observed benefit to a ‘generation gap’ between trials of ACEis and ARBs [9]. The primary ARB trials were performed a decade following the seminal ACEis trials, during which there was greater availability of evidence-based primary and secondary prevention strategies, and coronary revascularisation techniques. However, treatment-related reductions in BP would still be expected to translate into reduced incidence of MI and death, independent of background therapy and the contemporaneous standard of care. Although head-to-head trials comparing ACEis and ARBs represent the only truly objective means by which to assess the ARB-MI paradox, there exists a paucity of such high-quality clinical trial and meta-analysis data. In the most recent meta-analysis of head-to-head ACEi-ARB trials, which included 5 trials of 22,542 patients without heart failure, the risk of MI with ARBs was not significantly different than with ACEis (RR 1.07, 95% confidence intervals, CI 0.94–1.22) [10]. The risk of all-cause mortality, in 7 studies, was similar between ARB and ACEi groups (RR 0.98, 95% CI 0.90–1.07). The majority of patients included in these analyses (~76%) originated from the ONTARGET study [16]. Despite a marked ARB-associated reduction in systolic BP (expected to translate into a risk reduction of up to 5%), ONTARGET reported no significant associated benefit of ARB over ACEi.

Evaluation of the association between treatment-associated BP reduction and incidence of clinical outcomes will provide greater insight into the mechanism of the ARB-MI paradox, and advance understanding of the comparative safety and efficacy of ACEis and ARBs.

## Objectives

We aim to perform a systematic review and meta-analysis, of all available ACEi and ARB randomised controlled trial data. Analysis will be performed using summary-level data. Specifically, we will (i) investigate the extent to which the reported clinical outcomes in randomised trials of ACEis and ARBs are attributable to changes in systolic BP, using meta-regression, and (ii) ascertain if either class of drug has activity independent of BP reduction. We will calculate effect sizes, standardised per 10 mmHg difference in systolic BP, for each study outcome, where a BP-dependent effect is observed (see below).

## Methods

This protocol is in compliance with the relevant extension of the Prospective Reporting Instructions for Systematic Review and Meta-Analysis Statement; PRISMA-P (see Additional file 1) [17]. Final reporting of this study will be compliant with the main PRISMA statement [18]. This study is registered on PROSPERO, an international register of systematic reviews (CRD42017072988) [19]. This project was exempt from formal institutional ethical review. A summary of the key elements of the design of this study (PICO; population, intervention, comparison and outcome) is presented in Table 1.

**Table 1:** A summary of the main study elements in PICO format

Study elements	Description
Participants	Patients with an indication for inhibition of the renin-angiotensin aldosterone system, stratified into those with (i) hypertension and (ii) heart failure.
Intervention	Angiotensin II receptor blockers (ARBs) and angiotensin-converting enzyme inhibitors (ACEis)
Control or comparison	Placebo, or other classes of antihypertensive agent
Outcome	Standardisation will be performed to estimate the effect of a 10 mmHg reduction in blood pressure with each class of drug on the relative risk of each outcome. Primary: myocardial infarction and all-cause mortality Secondary: cardiovascular mortality, stroke, heart failure and revascularization

### Eligibility criteria

The study inclusion criteria are (i) randomised, placebo or active comparator-controlled studies, (ii) use of a prospective, randomised, open, blinded end-point (PROBE) design [20], (iii) trials evaluating at least one drug of the ACEi class (benazepril, captopril, cilazapril, delapril, enalapril, fosinopril, imidapril, lisinopril, moexipril, perindopril, quinapril, ramipril, spirapril, temocapril, trandolapril or zofenopril) or the ARB class (azilsartan, candesartan, eprosartan, fimasartan, irbesartan, losartan, olmesartan, telmisartan or valsartan), administered orally, (iv) investigator-choice of specific comparator agent (within a drug class), (v) any indication for ACEi or ARB therapy (hypertensive and non-hypertensive participants), (vi) human, phase II, III or IV studies, reporting clinical outcomes, (vii) studies reporting at least 500 patient-years of prospective follow-up in each study arm and (viii) studies of combination drug regimens, in which one of the regimens includes an ACEi or ARB. No date restrictions will be applied. No language restrictions will be applied; translation will be sought where necessary. Conference abstracts and other so-called ‘grey literature’ will only be included if a corresponding full, peer-reviewed publication is identifiable.

The study exclusion criteria are (i) studies that do not report relevant clinical endpoints (at least one of MI, all-cause mortality, stroke, heart failure, revascularisation or cardiovascular mortality), (ii) studies in which the control group received no treatment (i.e. neither placebo, nor active treatment), (iii) studies retracted subsequent to initial publication and (iv) lack of sufficient data to calculate between-group differences in systolic BP.

Trials that predominantly recruit participants with heart failure (HF) at baseline *will* be included in this meta-regression study. Previous meta-regression analyses have also pooled data from both hypertension and HF studies of renin-angiotensin inhibition [21]. Given that hypertension is a leading risk factor for the development of HF and both frequently co-exist, we feel that the inclusion of both categories of trial is justified. Trials enrolling hypertensive patients (including those targeting non-HF populations) included significant number of patients with left ventricular impairment, and vice-versa. For example, of the 5193 patients that had ejection fraction measured in the HOPE study, 421 (8.1%) had an ejection fraction < 0.40 [22]. Furthermore, trials enrolling participants with HF generally have a lower between-group difference in blood pressure (from enrolment to follow-up), and greater event rate than trials of hypertensive participants [23]. Therefore, if a bias was introduced by the inclusion of trials recruiting subjects with heart failure, it would be towards the null.

All studies of the ACEi and ARB class, vs. placebo or active comparator, will be included in meta-regression analysis. Studies that report head-to-head comparison of ACEi and ARB will be excluded in sensitivity analysis, once in which the ACEi group is the control class, and once in which the ARB is the control class.

### Information sources

A pre-specified search strategy will be performed, querying the following bibliographic databases, from inception to the present date: (i) MEDLINE (NCBI PubMed; from 1946), (ii) ISI Web of Science (Clarivate Analytics, NY, USA; from 1900), (iii) EMBASE (Elsevier, Amsterdam, NL; from 1947) and (iv) the Cochrane Central Register of Controlled Trials (The Cochrane Collaboration, London, UK; from 1966). Unpublished trials will be identified using ClinicalTrials.gov (from 1997). Literature that has not undergone formal publication (‘grey literature’, including conference proceedings, dissertations and theses) will be identified using The Conference Proceedings Citation Index and the OpenGrey website (via http://www.opengrey.eu/). This will be used to identify peer-reviewed publications based on these data. Where the grey literature source is the only record of the execution of a study, it will not be included in the synthesis. The expected impact of the non-publication of such data will be qualitatively discussed, and (if examples are present), will be included as a sensitivity analysis. The reference lists of previous meta-analyses and clinical trials of the ACEi and ARB class will be retrieved and scrutinised to identify studies that may otherwise be overlooked (backward citation searching). Citation lists of included studies will also be checked (forward citation searching).

### Search strategy

A broad and comprehensive search strategy was developed following a scoping review of the topic area. The search strategy will include both free-text (title and abstract keyword) searching and controlled vocabulary searching (e.g. the MEDLINE Medical Subject Heading [MESH] terms). Terms will be grouped by two concepts: (i) drug identifier terms, including the names of individual agents (e.g. perindopril) and relevant drug classes (e.g. angiotensin-converting enzyme inhibitors) and (ii) clinical trial identifier terms (e.g. randomised, randomised). The search strategy was adapted from the Cochrane Highly Sensitive Search Strategy (sensitivity-maximising version) [24]. The primary MEDLINE search strategy is presented in Additional file 2. Searches with equivalent syntax have been adapted to query the other bibliographic databases described above. The search strategy will be performed centrally, querying all data sources on a single date. All search results will be exported and stored in eXtensible Markup Language (.xml) format. Duplicate records identified from multiple sources will be collapsed into a single, unique entry.

### Study records

#### Selection process

**Fig. 1:**
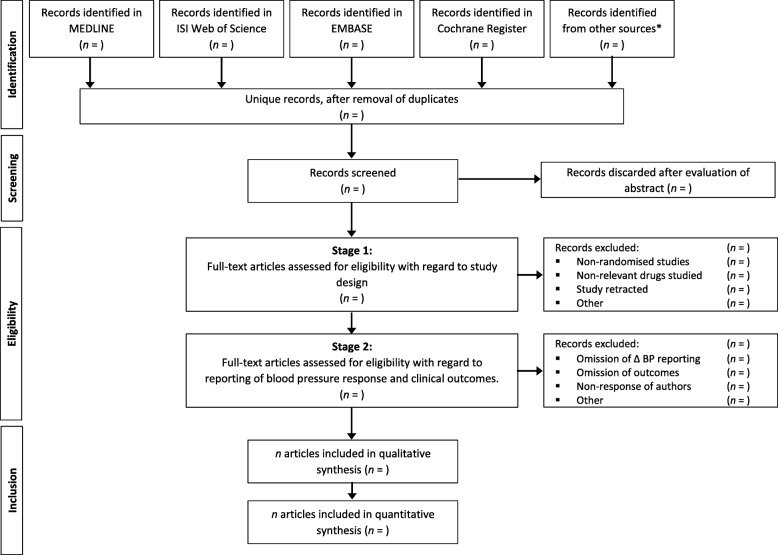
Preferred Reporting Items for Systematic Reviews and Meta-Analyses (PRISMA) flowchart depicting the proposed study selection process.*Other sources are described in full in the ‘Information sources’ section

Two independent reviewers will conduct a preliminary screen of all records identified by bibliographic searching; assessing each record for relevance according to pre-specified eligibility criteria. Studies that cannot be conclusively excluded by title and abstract screening will be advanced to full-text screening. Full-text articles will be retrieved in Portable Document Format (.pdf), and comprehensively reviewed using a 2-stage process. The first stage will assess the study with regard to design; the second stage will assess the study with regard to reporting of blood pressure differences and outcomes. Discrepancies between the two reviewers at any stage will be resolved through discussion, and if required, final arbitration will be performed by the senior authors (MHS and ASH). The flow of studies through the selection process, together with reasons for exclusion at the full-text stage, will be reported using a modified PRISMA diagram, as in Fig. 1.

#### Data management

Retrieval and storage of study records, abstracts and full-text articles will be performed using EndNote X8 (Thomson Reuters, NY, USA). The primary database will be stored on a server-based platform to enable real-time synchronisation of data between investigators.

#### Data extraction

Extraction of relevant data from included studies will be independently performed by JAB and MT. The concordance of each data point following extraction by both authors will be evaluated. Discrepancy will be resolved by discussion; final arbitration will be performed by MHS and ASH. A custom data extraction form will be piloted and optimised using a subset of five randomly selected studies satisfying the eligibility criteria. For those studies that satisfy the first stage of the eligibility screening process, but fail to report elements of necessary data (e.g. between-group differences in blood pressure, specific clinical endpoints) in the main study publication or supplementary appendices, the following steps will be taken: (i) searching for other peer-reviewed publications based on the primary study data, (ii) checking whether data are available in previous meta-analyses of this topic, (iii) searching for third-party sources of data, including US Food and Drug Administration (FDA) dockets, and, (iv) requesting data from the study author and/or sponsor. When data are not available from the primary study manuscript, a record of the data source will be made. If the minimum analytic dataset cannot be ascertained, such studies will be excluded. The expected effect of such exclusion (with regard to bias of the final analysis and results) will be summarised qualitatively in the final report.

When multiple publications arise from one study, relevant data will be extracted into a single form. Studies evaluating more than two groups, (e.g. ACEi vs. calcium channel blocker vs. placebo), are expected to occur infrequently in this context. For such studies, the group ‘shared’ in multiple comparisons (ACEi, in this example) and corresponding number of events will be equally divided, as outlined in the Cochrane Handbook for Systematic Reviews of Interventions [25]. This partially overcomes the unit-of-analysis (double-counting) error, although it is recognised that the resulting comparisons remain correlated. Sensitivity analyses, excluding such studies, will be performed to evaluate the extent of any bias that may be introduced.

#### Data items

**Fig. 2:**
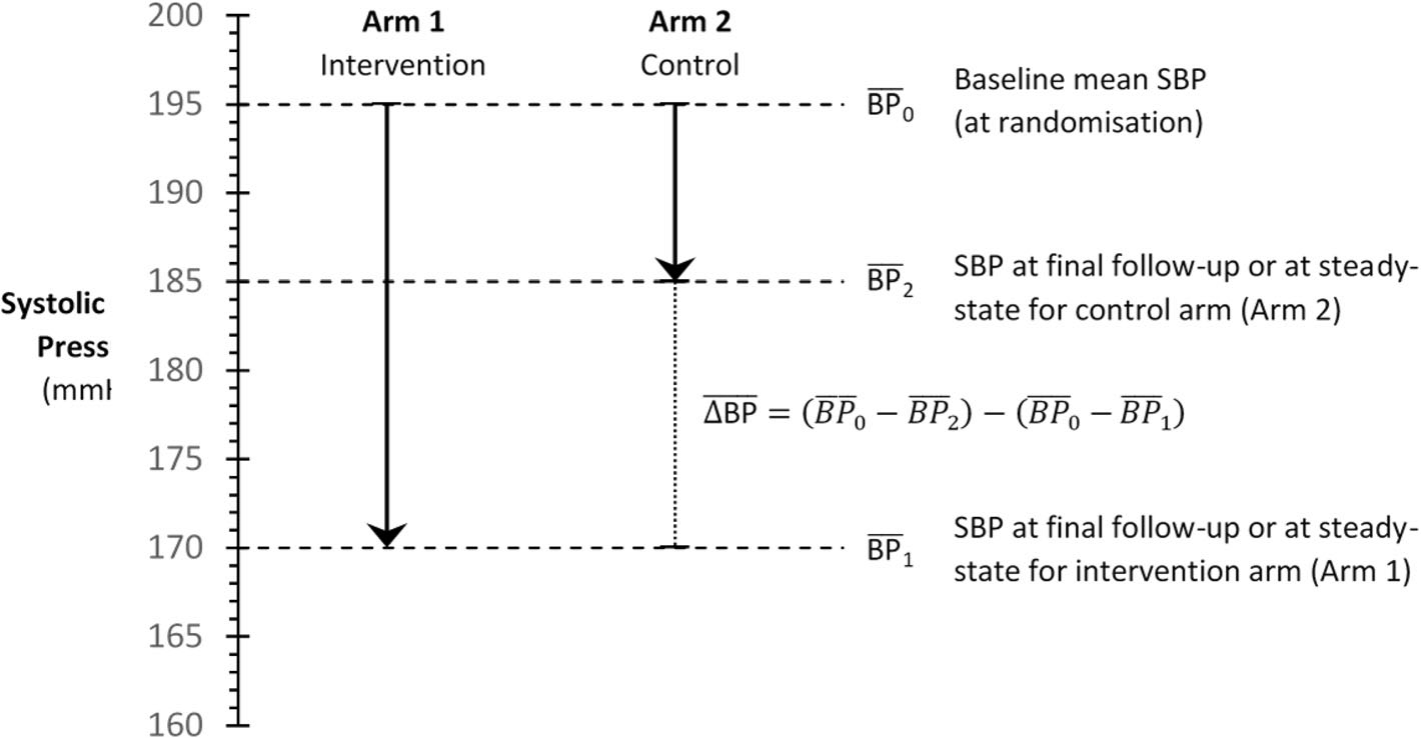
Schema illustrating calculation of mean between-group difference in blood pressure. Hypothetical data are presented. In this example, the mean between-group difference in blood pressure would be (195–185) − (195–170) = 10–25 = − 15 mmHg. That is, the intervention drug reduced blood pressure, on average, by 15 mmHg more than the control drug (placebo or active comparator)

All data items to be extracted are summarised in Table 2. Where conflicting, overlapping or duplicate study data are presented in multiple reports, only the most comprehensive or recent will be used, and the remainder discarded. Based on initial pilot analysis, the mean between-group difference in systolic BP during follow-up (or data required to robustly calculate between-group difference in systolic BP) is consistently well-reported in the relevant trials. Of 20 studies selected randomly that fulfil the proposed inclusion criteria, the mean between-group difference in BP was reported, or calculable, in 18 (90%). When not reported directly in the study manuscript, calculation will be performed. A schema for this measure is presented in Fig. 2. Reported differences in systolic BP will be extracted in preference to calculated differences; differences in systolic BP at the final study visit (from baseline) will be extracted in preference to mean or median differences during follow-up. However, where only average decreases in systolic BP are reported, these would be expected to result in bias towards the null, and therefore will be included. In the event that summary statistics other than mean and standard deviation are reported (e.g. median and interquartile range, confidence intervals), the recommended approach of Hozo et al. will be employed [26].

**Table 2:** Dictionary of items to be extracted from included studies, with description and rationale

Data item	Description and rationale
Study characteristics	
Study ID	Unique identifier for each study retrieved.
Trial acronym/first author	Either the trial acronym (if applicable, e.g. HOPE, VALUE) or first author of the primary study report if not (e.g. Smith AB et al.).
Year	Year of primary study publication.
Study design	Placebo-controlled, active comparator-controlled or PROBE design.
Duration of follow-up	Time period (in months) over which participants underwent follow-up
Age	Mean age of all patients included in analysis.
Sex	Number (%) of male study participants.
Hypertension	Number (%) of study participants with hypertension.
Heart failure	Number (%) of study participants with heart failure.
Diabetes	Number (%) of study participants with diabetes.
Ischaemic heart disease	Number (%) of study participants with ischaemic heart disease.
Loss to follow-up	Number (%) of study participants lost to follow-up
Revascularisation	Number (%) of study participants that underwent invasive coronary revascularisation
Concomitant drug therapy	Number (%) of study participants receiving other cardiovascular agents (β-blockers, calcium channel blockers, etc.)
Exposures and outcomes for each group	
Group class	Class of agent in the intervention and comparator arms (i.e. ACEi or ARB and placebo or active comparator).
Group agent	Identity of agent given to participants randomised to the intervention and comparator arms
*n* in group	Number in intervention and comparator arms (included in final analysis)
Baseline SBP	Baseline systolic blood pressure (mmHg) in each group. If multiple are provided (e.g. sitting, ambulatory, supine, etc.) sitting/clinic measurement will be used.
Follow-up SBP	Systolic blood pressure measured during follow-up in each group (mmHg). Any SBP measured at ‘steady-state’ during follow-up, or SBP averaged across visits may be included. If serial measurements are reported, the last measurement will be extracted. SBP after the first-dose of the medication will not be included.
Within-group difference in SBP	The within-group change in SBP from baseline to follow-up either directly reported or calculated as per Fig. 2. Directly reported differences will supersede calculated values.
Between-group difference in SBP	The between-group difference in SBP change, either directly reported or calculated as per Fig. 2. Directly-reported differences will supersede calculated values.
Baseline DBP	Baseline diastolic blood pressure (mm Hg) in each group. If multiple are provided (e.g. sitting, ambulatory, supine, etc.) sitting/clinic measurement will be used.
Follow-up DBP	Diastolic blood pressure measured during follow-up in each group (mm Hg). Any DBP measured at ‘steady-state’ during follow-up, or SBP averaged across visits may be included. If serial measurements are reported, the last measurement will be extracted. DBP after the first-dose of the medication will not be included.
Within-group difference in DBP	The within-group change in DBP from baseline to follow-up either directly reported or calculated as per Fig. 2. Directly-reported differences will supersede calculated values.
Between-group difference in DBP	The between-group difference in DBP change either directly reported or calculated as per Fig. 2. Directly reported differences will supersede calculated values.
All-cause mortality	The total number of deaths, of any cause, that occur in each group during follow-up. If the denominator is different to ‘*n* in group’, this must be specified.
Cardiovascular mortality	The number of cardiovascular deaths that occur in each group during follow-up. If the denominator is different to ‘*n* in group’, this must be specified.
Definition of cardiovascular mortality	Study definition of cardiovascular mortality, as per Table 2, below.
Myocardial infarction	The number of myocardial infarctions that occur in each group during follow-up. If the denominator is different to ‘*n* in group’, this must be specified.
Definition of myocardial infarction	Study definition of cardiovascular mortality, as per Table 2, below.
Stroke	The number of strokes that occur in each group during follow-up. If the denominator is different to ‘*n* in group’, this must be specified.
Definition of stroke	Study definition of stroke, as per Table 2, below.

Only data items for direct extraction are presented above. Calculation of group specific event rates, etc. will be performed during the analytic phase of the study. *PROBE* prospective randomised open-blinded end-point study design

Pilot analysis has suggested that the expected range of between-group differences in systolic blood pressure lies between 5 and − 20 mmHg (maxima and minima, respectively), with an expected standard deviation of approximately 5 mmHg. This degree of variability approximates well with that observed in a previous meta-analysis [21].

#### Outcomes

The primary outcomes will be MI (non-fatal and fatal, including sudden death) and all-cause mortality (death from any cause). These outcomes were chosen as the focus of current inquiry as they represent the source of persisting controversy, and are measured most consistently across studies. Secondary outcomes were cardiovascular mortality (defined as per study; often a composite of fatal myocardial infarction, stroke, peripheral vascular disease and sudden death), stroke (non-fatal and fatal), heart failure requiring hospitalisation, and unplanned revascularisation. The number of events occurring in the total at-risk population (using intention-to-treat analysis, if reported) will be extracted. This will be used to calculate a risk ratio for each study, which will be reported in addition to the raw data.

When these endpoints are not reported verbatim in studies (e.g. only fatal MI is reported), this will be included, with appropriate acknowledgement in the final report. The hierarchy for reporting non-specific outcomes is presented in Table 3.

**Table 3:** The hierarchy of outcomes to be extracted and included in final data synthesis

Outcome	Status	Rank	Explanation
All-cause mortality	Primary	1	Total number of deaths in each study group due to any attributed cause.
Myocardial infarction	Primary	1	Non-fatal or fatal myocardial infarction, or sudden cardiac death
		2	Non-fatal or fatal myocardial infarction
		3	Fatal myocardial infarction, or sudden cardiac death
		4	Non-fatal myocardial infarction only
		5	Fatal myocardial infarction only
		6	Any other definition of a myocardial infarction-related endpoint
Cardiovascular mortality	Secondary	1	Fatal myocardial infarction, stroke, other cardiovascular disease or sudden death
		2	Fatal myocardial infarction, stroke or other cardiovascular disease
		3	Fatal myocardial infarction, stroke or sudden death
		4	Fatal myocardial infarction or stroke
		5	Any other definition of cardiovascular mortality
Stroke	Secondary	1	Non-fatal or fatal stroke, not including transient ischaemic attack
		2	Fatal stroke
		3	Non-fatal stroke
		4	Non-fatal stroke or transient ischaemic attack
		5	Non-fatal or fatal stroke or transient ischaemic attack
		6	Any other definition of a stroke-related endpoint
Revascularisation	Secondary	1	Any need for unplanned revascularisation
Heart failure	Secondary	1	Management of heart failure, requiring medical attention, hospital attendance or admission

Rank 1 is the preferred outcome definition to be extracted and included in data synthesis. In the event of a study not reporting the first-ranked outcome, and this data not being available from contacting authors and sponsors, the next lowest ranked endpoint reported (or calculable without double-counting) in each study will be included in final data synthesis

Where few outcomes are observed, resulting in a divide-by-zero error and unspecified risk ratio, one outcome will be added to each cell, in order to permit inclusion of the study. For example, in a hypothetical trial in which there were three deaths in the control arm and none in the active comparator arm, it would be impossible to calculate a risk ratio. The addition of one event to each cell of the contingency table would permit estimation of the relevant ratio measures.

#### Risk of bias in individual studies

The risk of bias will be ascertained by two reviewers in parallel, using The Cochrane Risk of Bias Tool [27]. Assessment will be performed at the study level, and will focus on selection, performance, detection, attrition and reporting biases. The risk of bias for each included study will be taken into consideration during data synthesis. Sensitivity analysis, excluding those studies at greatest risk of bias, will be performed. In addition, the Grades of Recommendation, Assessment, Development, and Evaluation (GRADE) system will be used to summarise the quality of evidence, for each outcome [28].

#### Data synthesis

Studies will be included in qualitative and quantitative synthesis if they fulfil all eligibility criteria. Notable records discarded during the screening procedure will be discussed, particularly if these required arbitration, or have been specifically included in or excluded from previous meta-analyses. Study characteristics will be summarised using means and standard deviations (or medians and interquartile ranges) for continuous variables, and numbers and percentages for categorical variables. A narrative report of study characteristics will also be provided.

Between-study heterogeneity will be estimated characterised using the *I*^2^ statistic and quantified using Cochran’s *Q* statistic. Sources of heterogeneity, derived from the clinical characteristics of patients enrolled to each study (Table 2) will be examined. In the absence of significant between-study heterogeneity, inverse variance-weighted fixed-effects meta-analysis will be performed. Random-effects meta-analysis, using the methodology of DerSimonian and Laird, will be performed as a sensitivity analysis [29]. The pooled effect estimate (relative risk, RR) will be reported with corresponding 95% confidence intervals and *p* values. All *p* values will be calculated using two-tailed tests, with type I error of 0.05. Additionally, standardisation will be performed to estimate the pooled effect of a 10 mmHg reduction in blood pressure with each class of drug on the relative risk of each outcome, with corresponding 95% confidence intervals. This will be performed using the methodology of Tierney et al. using the formula below [30, 31].

$$ {\mathrm{RR}}_{\mathrm{standardised}}={e}^{10\frac{\sum \Delta  {\mathrm{SBP}}_i\bullet {W}_i\bullet \log \left({\mathrm{RR}}_i\right)}{\sum \Delta  {{\mathrm{SBP}}_i}^2\bullet {W}_i}} $$

Where ∆SBP_i_ is the between-group difference in systolic BP, log (RR_i_) is the natural log-transformed relative risk for each outcome and *W*_i_ is the inverse variance, for each trial, *i*. Meta-regression will be performed to identify the impact of blood pressure reduction as a moderator of clinical outcome, and evaluate the assumption that reductions in RR will be proportional to the achieved reduction in BP. Meta-regression plots, with 95% confidence intervals, will be plotted by drug class and outcome. Relative risk will be plotted against between-group reduction in systolic BP. The *x*-axis intercept will be examined to assess the blood pressure-dependency of the effect of each agent (i.e. a reduction in clinical endpoints at a between-group difference in BP of 0 mmHg implies pharmacological activity independent of BP reduction). Subgroup analysis will be performed to address the presence of interaction with (i) overall duration of study follow-up, (ii) percentage usage of other agents (β blockers and calcium channel blockers), (iii) percentage of study participants revascularised, (iv) year of study publication and (v) enrolment period of patients; each categorised appropriately. *p* values for interaction will be presented for each case. The presence of meta-bias (specifically, publication bias) will be examined by visual inspection of Funnel plots and calculation of Egger’s statistic, for both ACEi and ARB trials. All analyses will be performed using Stata (version 14.0; StataCorp, College Station, TX, USA) and *R* (version 3.4.1; The R Foundation for Statistical Computing, Vienna, Austria). Narrative evaluation of the risk of bias in individual studies will be undertaken to suggest the overall strength of the body of evidence.

#### Sensitivity analyses

All studies of the ACEi and ARB class, vs. placebo or active comparator, will be included in the primary meta-regression analysis. Multiple sensitivity analyses will be performed to evaluate restriction of analysis to exclude studies with (a) open-label (e.g. PROBE) design, (b) head-to-head comparison of ACEi and ARB only, (c) high-risk of bias, (d) greater than two randomisation arms and (e) principal recruitment of participants with HF. Both fixed effects (inverse variance-weighted) and random effects (DerSimonian and Laird-weighted) will be performed.

## Discussion

The existence and mechanism of an ARB-MI paradox is controversial. We envisage that this systematic review will provide a detailed, state-of-the-art and unbiased synthesis of the evidence regarding the relationship between clinical outcomes and BP reduction as a result of treatment with ACE inhibitors and ARBs. To date, several methodologically similar meta-regression analyses have been performed. One such analysis included only those trials participating in the Blood Pressure Lowering Treatment Trialists’ Collaboration; lacking a systemic methodological rigour [21]. A search strategy, study inclusion and exclusion criteria were not defined on an a priori basis. Furthermore, this study was likely underpowered, in part due to the era in which it was performed (pre-2005). As such, this study is unlikely to represent an unbiased representation of all available clinical evidence. Emergence of further relevant studies in the intervening period may enable sufficient power to form additionally robust conclusions. Further analyses, not restricted to the ACEi and ARB class, report a consistent relationship between reductions in BP and cardiovascular risk reduction [5, 32]. However, between-class ACE-ARB comparisons were not the major focus of such studies [33].

Previous meta-regression analyses (such as that by Messerli et al. [10]) estimated whether ACEi or ARB treatment effects (on a relative risk scale) were associated with the underlying baseline risk, as measured by the event rate observed in the control group. This is a flawed approach, which produces misleading results [34, 35]. Specifically, the independence assumption of regression is invalidated; the control event rate is included in the denominator of the relative risk estimate. In the absence of any true association, such analyses will always determine that an observed benefit will be more pronounced in trials with high control group event rates, than trials with low control group event rates; a self-fulfilling prophecy [36].

Greater characterisation of the relationship between drug-mediated BP reductions and clinical outcomes may better guide utilisation of ACE inhibitors and ARBs, and may highlight the need for further analysis, using individual participant-level randomised trial data.

## Additional files

Additional file 1: PRISMA-P 2015 Checklist. (DOCX 30 kb)

Additional file 2: Primary MEDLINE (PubMed) search strategy. (DOCX 14 kb)

## Abbreviations

ACEi: Angiotensin-converting enzyme inhibitor
ARB: Angiotensin receptor blocker
AT: Angiotensin II receptor
BP: Blood pressure
BPLTTC: Blood Pressure Lowering Treatment Trialists’ Collaboration
FDA: Food and Drug Administration
MI: Myocardial infarction
