# Supplementary material for: Blood pressure reduction and clinical outcomes with angiotensin-converting enzyme inhibitors and angiotensin II receptor blockers: protocol for a systematic review and meta-regression analysis
Source: Syst Rev. 2018 Aug 25;7:131. doi: 10.1186/s13643-018-0779-5 (PMC6109343; doi:10.1186/s13643-018-0779-5)
Supplement: Supplementary file 2 — Primary MEDLINE (PubMed) search strategy. (DOCX 14 kb) [file 13643_2018_779_MOESM2_ESM.docx]

**Additional file 2**

Primary MEDLINE (PubMed) search strategy:

(((("angiotensin-converting enzyme inhibitors"[MeSH] OR "angiotensin receptor antagonists"[MeSH] OR "angiotensin II type 1 receptor blockers"[MeSH] OR "renin-angiotensin system"[MeSH] OR "receptors, angiotensin"[MeSH] OR "angiotensin-converting enzyme inhibitors"[Pharmacological Action] OR "angiotensin receptor antagonists" [Pharmacological Action] OR (("renin-angiotensin"[All Fields] AND "system"[All Fields]) OR "renin-angiotensin system"[All Fields] OR ("renin"[All Fields] AND "angiotensin"[All Fields] AND "system"[All Fields]) OR "renin angiotensin system"[All Fields] OR ("renin"[All Fields] AND "angiotensin"[All Fields] AND "aldosterone"[All Fields] AND "system"[All Fields]) OR "renin angiotensin aldosterone system"[All Fields] OR ("angiotensin-converting"[All Fields] AND "enzyme"[All Fields]) OR "angiotensin-converting enzyme"[All Fields] OR ("angiotensin"[All Fields] AND "converting"[All Fields] AND "enzyme"[All Fields]) OR "angiotensin converting enzyme"[All Fields] OR ("angiotensin"[All Fields] AND "receptor"[All Fields]) OR "angiotensin receptor"[All Fields]) AND ("blocker"[All Fields] OR “blockage”[All Fields] OR “antagonist”[All Fields] OR “antagonism”[All Fields] OR “inhibitor”[All Fields] OR “inhibition”[All Fields])) OR (("alacepril”[Supplementary Concept] OR "alacepril"[All Fields]) OR ("benazepril"[Supplementary Concept] OR "benazepril"[All Fields]) OR ("captopril"[MeSH] OR "captopril"[All Fields]) OR ("cilazapril"[MeSH] OR "cilazapril"[All Fields]) OR ("delapril"[Supplementary Concept] OR "delapril"[All Fields]) OR ("enalapril"[MeSH] OR "enalapril"[All Fields]) OR ("fosinopril"[MeSH] OR "fosinopril"[All Fields]) OR ("imidapril"[Supplementary Concept] OR "imidapril"[All Fields]) OR ("lisinopril"[MeSH] OR "lisinopril"[All Fields]) OR ("moexipril"[Supplementary Concept] OR "moexipril"[All Fields]) OR ("perindopril"[MeSH] OR "perindopril"[All Fields]) OR ("quinapril"[Supplementary Concept] OR "quinapril"[All Fields]) OR ("ramipril"[MeSH] OR "ramipril"[All Fields]) OR ("spirapril"[Supplementary Concept] OR "spirapril"[All Fields]) OR ("temocapril hydrochloride"[Supplementary Concept] OR "temocapril hydrochloride"[All Fields] OR "temocapril"[All Fields]) OR ("trandolapril"[Supplementary Concept] OR "trandolapril"[All Fields]) OR ("zofenopril"[Supplementary Concept] OR "zofenopril"[All Fields] OR ("azilsartan"[Supplementary Concept] OR "azilsartan"[All Fields]) OR ("candesartan"[Supplementary Concept] OR "candesartan"[All Fields]) OR ("eprosartan"[Supplementary Concept] OR "eprosartan"[All Fields]) OR ("fimasartan"[Supplementary Concept] OR " fimasartan"[All Fields]) OR ("irbesartan"[Supplementary Concept] OR "irbesartan"[All Fields]) OR ("losartan"[MeSH] OR "losartan"[All Fields]) OR ("olmesartan"[Supplementary Concept] OR "olmesartan"[All Fields]) OR ("telmisartan"[Supplementary Concept] OR "telmisartan"[All Fields]) OR ("valsartan"[Supplementary Concept] OR "valsartan"[All Fields]))) AND (“clinical trial”[Publication Type] OR “meta-analysis”[Publication Type] OR "clinical trials as topic"[MeSH] OR “trial”[ti] OR “randomised”[tiab] OR “randomized”[tiab] OR “randomly”[tiab] OR “placebo”[tiab])) AND ("humans"[MeSH]))) NOT ("review"[Publication Type])
